# Analyzing Roles of NUSAP1 From Clinical, Molecular Mechanism and Immune Perspectives in Hepatocellular Carcinoma

**DOI:** 10.3389/fgene.2021.689159

**Published:** 2021-07-20

**Authors:** Wenjie Zhu, Jian Xu, Zehao Chen, Jianxin Jiang

**Affiliations:** Department of Hepatobiliary Surgery, Renmin Hospital of Wuhan University, Wuhan, China

**Keywords:** hepatocellular carcinoma, NUSAP1, GEO, ICGC, TCGA, cell cycle, immune, clinical

## Abstract

Hepatocellular carcinoma (HCC) is one of the most common carcinomas worldwide. Our study aims to analyze how NUSAP1 affects progression of HCC from clinical, molecular mechanism and immune perspectives. Firstly, we downloaded GSE62232, GSE102079, GSE112790, and GSE121248 gene expression profile datasets from GEO database. R studio was used to screen DEGs of each dataset, and 86 overlapping DEGs of the four datasets were screened at last. Then, CytoHubba plug-in in Cytoscape software was used to screen out NUSAP1 from the 86 DEGs. Subsequently, survival analysis, clinical correlation analysis, independent prognostic analysis, and GSEA enrichment analysis of NUSAP1 were analyzed using HCC patients from GSE76427 dataset, ICGC database, and TCGA database. The results revealed that HCC patients with higher expression level of NUSAP1 had a worse prognosis. NUSAP1 was an independent prognostic factor of HCC, and it may promote HCC progress by regulating cell cycle. To further elucidate its underlying molecular mechanism, we used cBioProtal online data analysis tool to screen all co-expression genes of NUSAP1 and used top 300 co-expression genes to accomplish KEGG and GO enrichment analysis; the results confirmed that NUSAP1 accelerated progression of HCC by regulating cell cycle. We continued to draw KEGG pathway map of cell cycle using co-expression genes enriched in cell cycle pathway by KEGG online tool. The map depicted that most of co-expression genes of NUSAP1 were located in S phase and G2/M phase of the cell cycle, and they could regulate the genes in G1 phase. To further understand the mechanism of cell cycle, we also did qRT-PCR, Western blot, and flow cytometry; the results showed that NUSAP1 was closely associated with CDK4, CDK6, and cyclinD1, which could regulate G1 to S phase transition. Besides, we also analyzed correlation between NUSAP1 and immune cells using HCC patients from GSE76427 dataset, ICGC database, and TCGA database. NUSAP1 was associated with some immune cells, and we speculated that NUSAP1 could also promote HCC progression by influencing T cell CD4 memory resting and macrophage M0 through some underlying mechanism.

## Introduction

Hepatocellular carcinoma (HCC) is the sixth most common and the fourth deadliest malignant tumors globally (Bray et al., 2018). It is particularly prevalent in China, Asia, and Africa (Forner et al., 2012). About 60% new HCC cases occur in China every year, and the 5-year survival rate is approximately 12% (Chen et al., 2016; Zeng et al., 2018). In terms of etiology, the occurrence of HCC is related to HBV, HCV, alcohol, aflatoxin, autoimmune diseases, diabetes, obesity, and so on (Bruix and Sherman, 2011; Villanueva, 2019). There are many treatment options for HCC now, including surgical resection, orthotopic liver transplantation, ablation, transcatheter arterial chemical embolization (TACE), and systemic chemotherapy (Cillo et al., 2014; Xue et al., 2015; Marrero et al., 2018). Despite so many treatment options available, the overall survival (OS) of HCC patients remains poor due to its extremely high rates of postoperative recurrence and metastasis (Villanueva et al., 2011). Therefore, there is an urgent need to investigate underlying molecular mechanism of HCC occurrence, development, and poor prognosis in order to explore better strategies of prevention, diagnosis, and treatment in HCC.

At present, bioinformatics methods and microarrays have been widely used to screen differentially expressed genes (DEGs) in tumors. Herein, we collected four HCC-related mRNA microarray datasets from Gene Expression Omnibus (GEO) and obtained DEGs between HCC and normal liver tissues by R studio. Then, we obtained overlapping DEGs of the four HCC-related mRNA microarray datasets by Venn online tool and analyzed the relationships among overlapping genes by protein–protein interaction (PPI) network. MCODE and ctyoHubba plug-in in Cytoscape software were respectively used to screen the most significant module in PPI network and key gene in all overlapping genes. GEO database, ICGC database, and TCGA database were used to explore the relationship between the key gene and HCC patients’ survival and prognosis. Analyzing multiple databases, we explored the underlying mechanism by which key gene influences progression of HCC. Although our study was a bioinformatic-based analysis, it may be of significant value to studies in regard to explore clinical and molecular mechanism of HCC in the future.

## Materials and Methods

### Microarray Data

In our study, four gene expression profile datasets were screened using the GEO^1^ database of NCBI, including the GSE62232 series (Schulze et al., 2015), the GSE102079 series (Chiyonobu et al., 2018), the GSE112790 series (Shimada et al., 2019), and the GSE121248 series (Wang et al., 2007). The GSE62232 dataset included 81 HCC tissue samples and 10 normal liver tissue samples, the GSE102079 dataset included 183 HCC tissue samples and 15 normal liver tissue samples, the GSE112790 dataset included 70 HCC tissue samples and 37 normal liver tissue samples, and the GSE121248 dataset included 152 HCC tissues samples and 105 adjacent normal liver tissue samples. The microarray data from GSE62232, GSE102079, GSE112790, and GSE121248 were based on the GPL570 platform (HG-U133_Plus_2) Affymetrix Human Genome U133 Plus 2.0 Array.

### Identification of DEGs

R studio was used to identify DEGs between HCC and normal liver samples by analyzing GSE62232, GSE102079, GSE112790, and GSE121248 raw data of CEL files. Firstly, we used the RMA package to normalize all the raw database and the Affy package to assess the quality of samples in each dataset. Then, according to the annotation information in GPL570 platform, probes were changed into the corresponding gene symbols using R studio. At last, the Limma package was used to identify DEGs. The criterion of identifying DEGs was | logFC| > 2 and adjusted *p* < 0.05.

### Screening Overlapping DEGs

In order to screen overlapping DEGs among GSE62232, GSE102079, GSE112790, and GSE121248, we use R studio to draw a Venn diagram and find the overlapping DEGs among GSE62232, GSE102079, GSE112790, and GSE121248.

### Construction and Module Analysis of the Protein–Protein Interaction (PPI) Network

The PPI network was generated by the Search Tool for the Retrieval of Interacting Genes (String^2^, Ver-sion:11.0) online database (Szklarczyk et al., 2015). The MCODE plug-in in Cytoscape software was used to select significant modules in the PPI network, and we could find the most important module in all selected significant modules. The criteria of selecting significant modules were degree cutoff = 2, node density cutoff = 0.1, node score cutoff = 0.2, k-core = 2, and maximum depth = 100.

### Hub Gene Selection and Analysis

CytoHubba plug-in in Cytoscape software was used to screen the top 10 hub genes in PPI network. There were 12 calculating methods in cytoHubba, which included Betweenness, BottleNeck, Closeness, ClusteringCoefficient, Degree, DMNC, EcCentricity, EPC, MCC, MNC, Radiality, and Stress. We used the 12 different calculating methods to get 12 different outcomes of the top 10 hub genes. By analyzing the 12 outcomes, we screened hub gene NUSAP1.

### Acquisition of Clinical Data and Gene Expression Files

We downloaded clinical data and gene expression files from GSE76427 dataset, International Cancer Genome Consortium database (ICGC)^3^, and The Cancer Genome Atlas Program (TCGA)^4^ database. The GSE76427 dataset included 52 normal liver samples’ gene expression levels and 115 HCC samples’ gene expression levels and clinical information. The ICGC database contained 202 normal liver samples’ gene expression levels and 243 HCC samples’ gene expression levels and clinical information. The TCGC database contained 50 normal liver samples’ gene expression levels and 374 HCC samples’ gene expression levels and clinical information.

### Survival Analysis, Clinical Correlation Analysis, Independent Prognostic Analysis, and Immune Correlation Analysis

We used R studio to accomplish survival analysis, clinical correlation analysis, independent prognostic analysis, and immune correlation analysis using HCC patients’ gene expression files and clinical information from GSE76427 dataset, ICGC database, and TCGA database. The survival package was used to accomplish survival analysis and independent prognostic analysis. The Ggpubr package was used to do clinical correlation analysis. Limma, corrplot, vioplot, ggplot2, ggpubr, ggExtra, and VennDiagram packages were used to achieve outcomes of immune correlation analysis.

### RNA Isolation and Quantitative Real-Time PCR

Prior to the qRT-PCR proceedings, total RNA was extracted using TRIzol reagent (Thermo Fisher Scientific, Inc.). cDNA was obtained by reverse transcription using Thermo Fisher Scientific reagent Kit. qRT-PCR was conducted by a Bio-Rad Real-Time PCR Detection System (Bio-Rad Laboratories, Inc., Hercules, CA, United States). The following primers were used: NUSAP1 forward, 5′-AGCCCATCAATAAGGGAGGG-3′ and reverse, 5′-ACCTGACACCCGTTTTAGCTG -3′.

### siRNA Treatments

siRNA duplexes against NuSAP1 were transfected into HCC cancer cells using Lipofectamine 2000 (Invitrogen). The sequences of siRNA duplex sense were as shown below:

si1: 5′-ATAAGCGTTCACTGACCAA-3′

si2: 5′-CCTTAAAGCTCAAATTCTT-3′

si3: 5′-TTCTCCGAACGTGTCACGT-3′

### Cell Cycle Analysis

One day prior to transfection, HepG2 and Huh-7 cells were seeded into six-well plates. After successful transfection, cells were washed using PBS and fixed in 75% ice-cold alcohol. Before analysis, the cells were incubated in 500 μl sample buffer [50 μg/ml propidium iodide (Merck KGaA, Darmstadt, Germany) and 0.25 mg/ml of RNase A (Takara Bio, Inc., Otsu, Japan)] for 30 min at room temperature. The numbers of cells in each phase were detected using a BD FACSCalibur Flow Cytometer (BD Biosciences, San Jose, CA, United States) and analyzed by FlowJo software (FlowJo, LLC, Ashland, OR, United States).

### Western Blot

Before Western blot was carried out, the whole-cell protein lysates were extracted using radioimmunoprecipitation assay buffer (Beyotime Institute of Biotechnology, Shanghai, China). Following, protein lysates were separated by SDS-PAGE and blotted onto PVF membranes (Bio-Rad). After blocking, the membranes were incubated with NUSAP1 antibody, CDK4 antibody, CDK6 antibody, cyclinD1 antibody, and GAPDH antibody (Sangon Biothch, Shanghai) overnight at 4°C. Next, the membranes were probed with secondary HRP Linked Secondary Antibodies (Sangon Biothch, Shanghai). Finally, immunoblots were visualized by imaging systems (DenvilleScientific Inc., Holliston, MA, United States).

### Go and KEGG Enrichment Analysis

Firstly, we used cBioPortal^5^ (Cerami et al., 2012; Gao et al., 2013) to screen all co-expression genes of NUSAP1. Subsequently, we used DAVID^6^ (Huang et al., 2007, 2009) to generate KEGG pathway analysis of the top 300 co-expression genes of NUSAP1. The pathway map of cell cycle was generated by Kyoto Encyclopedia of Genes and Genomes (KEGG)^7^ (Ogata et al., 1999), and the corresponding positions of co-expression genes in the map were marked in red color. Next, we continued to use DAVID to get GO analysis results of the top 300 co-expression genes, which included biological process (BP), cellular component (CC), and molecular function (MF). In addition, to visualize the results of GO and KEGG analysis, we also used the top 300 co-expression genes to draw bar plots and bubble plots of GO and KEGG analysis using ggplot2, enrichplot, org.Hs.eg.db, and clusterProfiler packages in R studio.

### GSEA Enrichment Analysis

GSEA software was used to generate enrichment analysis results by comparing NUSAP1 high expression group and NUSAP1 low expression group in KEGG and GO gene sets. GSEA software was downloaded from the Broad Institute^8^ (Subramanian et al., 2005). Besides, we used plyr, ggplot2, grid, and gridExtra packages in R studio to combine our GSEA enrichment analysis results in a single graph for easy comparison.

### Immunophenoscore (IPS) Analysis

IPS was composed of effector cells, immunosuppressive cells, MHC molecules, and immunomodulators, and it was calculated based on the gene expression in representative cell types. The IPS of HCC patients was downloaded from The Cancer Immunome Atlas (TCIA).^9^

### Statistical Analysis

GraphPad Prism (version: 8.0.1) and R studio (version: 4.0.3) were used for data analyses; the data was considered to be significant when *p* < 0.05.

## Results

### Identification and Analysis of DEGs

**FIGURE 1:**
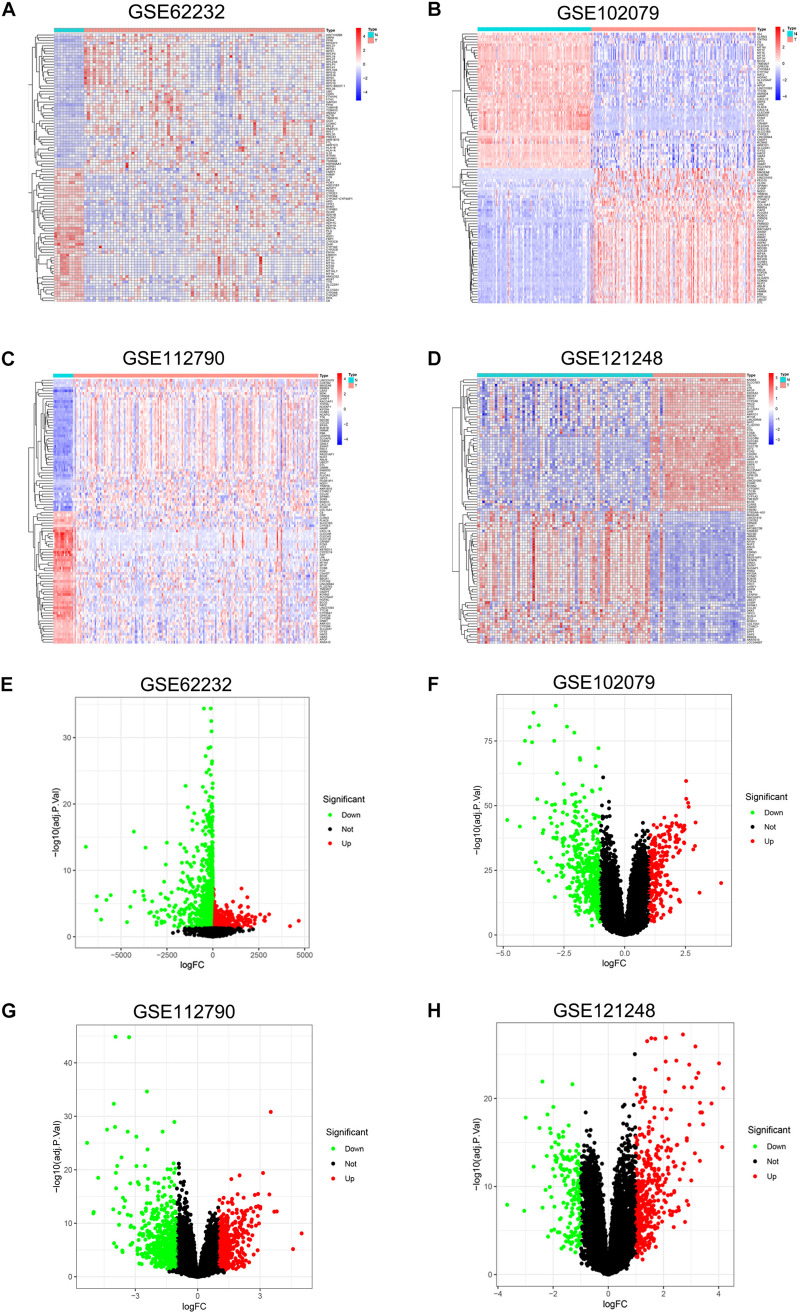
Heatmap and volcano plot of DEGs in GSE62232, GSE102079, GSE112790, and GSE121248. **(A)** Heatmap of DEGs in GSE62232. **(B)** Heatmaps of DEGs in GSE102079. **(C)** Heatmaps of DGEs in GSE112790. **(D)**. Heatmaps of DGEs in GSE121248. **(E)** Volcano plot of DEGs in GSE62232. **(F)** Volcano plot of DEGs in GSE102079. **(G)** Volcano plot of DEGs in GSE112790. **(H)** Volcano plot of DEGs in GSE121248. In heatmaps, red color represented high expression level and blue color represent low expression level. In volcano plots, red dots were upregulated genes and green dots were downregulated genes.

**FIGURE 2:**
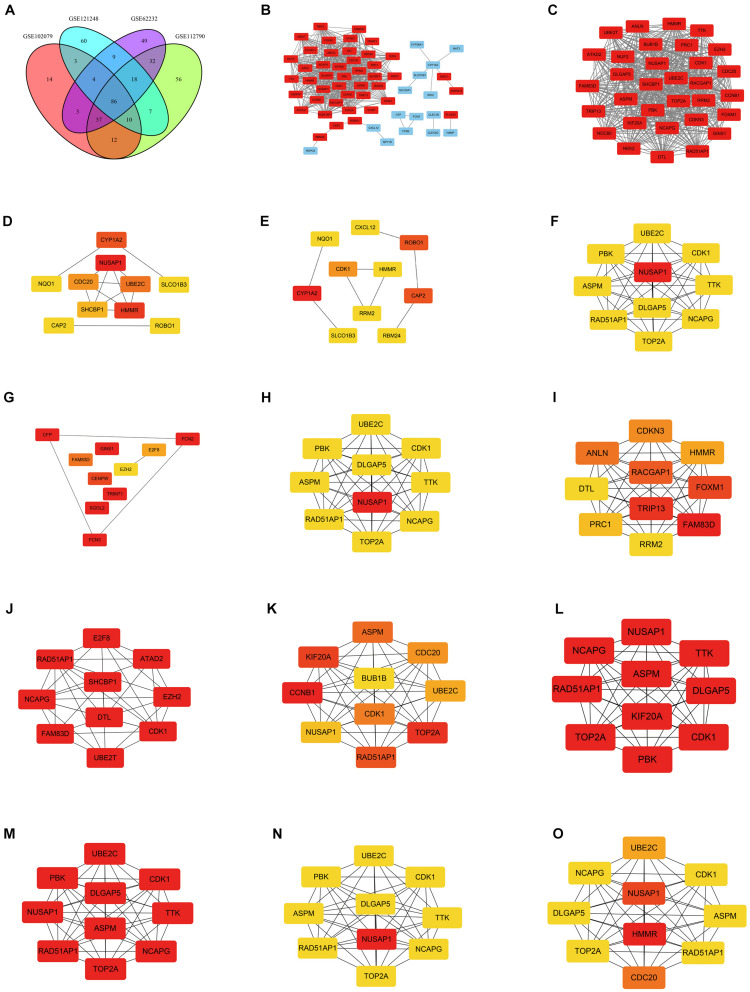
Venn diagram and PPI networks. **(A)** Identification of overlapping DEGs in GSE62232, GSE102079, GSE112790, and GSE121248; the four datasets shared 86 overlapping DEGs. **(B)** PPI network was constructed using 86 overlapping DEGs (including 55 upregulated genes and 31 downregulated genes). Upregulated and downregulated genes were marked in red and blue, respectively. **(C)** The most significant module of PPI network included 32 nodes and 474 edges. All genes in the most significant module were upregulated genes and marked in red. **(D)** Top 10 hub genes in the PPI network, which were calculated by method Betweenness. **(E)** Top 10 hub genes in the PPI network, which were calculated by method BottleNeck. **(F)** Top 10 hub genes in the PPI network, which were calculated by method Closeness. **(G)** Top 10 hub genes in the PPI network, which were calculated by method ClusteringCoefficient. **(H)** Top 10 hub genes in the PPI network, which were calculated by method Degree. **(I)** Top 10 hub genes in the PPI network, which were calculated by method DMNC. **(J)** Top 10 hub genes in the PPI network, which were calculated by method EcCentricity. **(K)** Top 10 hub genes in the PPI network, which were calculated by method EPC. **(L)** Top 10 hub genes in the PPI network, which were calculated by method MCC. **(M)** Top 10 hub genes in the PPI network, which were calculated by method MNC. **(N)** Top 10 hub genes in the PPI network, which were calculated by method Radiality. **(O)** Top 10 hub genes in the PPI network, which were calculated by method Stress. The darker the color, the higher the score.

DEGs of each microarray dataset were identified using R studio (238 in GSE62232, 258 in GSE102079, 169 in GSE112790, and 197 in GSE121248), and the cut-off criterion was | logFC| > 2 and adjusted *p* < 0.05. Heatmaps and volcano plots of DEGs in each microarray dataset were also draw by R studio and shown in Figure 1. Subsequently, we continued to screen common DEGs in four microarray datasets and 86 overlapping DEGs were identified; the results were shown in a Venn diagram (Figure 2A). Then, we used STRING online database to generate a PPI network of the 86 overlapping DEGs and used Cytoscape software for visualization (Figure 2B); it included 55 upregulated genes (marked in red) and 31 downregulated genes (marked in blue). MCODE plug-in in Cytoscape software was used to screen the most significant module in the PPI network of 86 DEGs (Figure 2C); it included 32 nodes and 474 edges, all of 32 genes in this module were upregulated genes (marked in red) in HCC tissues.

### Screening Out Key Gene NUSAP1

CytoHubba plug-in in Cytoscape software was used to find the top 10 hub genes in the PPI network containing 86 DEGs. There were 12 calculating methods in cytoHubba, which included Betweenness, BottleNeck, Closeness, ClusteringCoefficient, Degree, DMNC, EcCentricity, EPC, MCC, MNC, Radiality, and Stress. We used the 12 different calculating methods to get 12 different outcomes of the top 10 hub genes (Figures 2D–O). By analyzing the 12 different outcomes, we found that NUSAP1 existed in 8 outcomes and was the highest score in 6 outcomes; CDK1 existed in 9 outcomes and was the highest score only in 3 outcomes; RAD51AP1 existed in 8 outcomes and was the highest score only in 3 outcomes; NCAPG was shown in 8 outcomes and was the highest score only in 3 outcomes. ASPM was shown in 7 outcomes and was the highest score only in 2 outcomes; TOP2A was shown in 7 outcomes and was the highest score only in 3 outcomes; DLGAP5 was shown in 6 outcomes and was the highest score only in 2 outcomes; UBE2C was shown in 7 outcomes and was the highest score only in 1 outcome. Hence, we screened out NUSAP1 as our key gene and carried out our studies around it.

### Survival Analysis, Clinical Correlation Analysis, Independent Prognostic Analysis, and GSEA Enrichment Analysis of NUSAP1 by HCC Patients in GSE76427 Dataset

**FIGURE 3:**
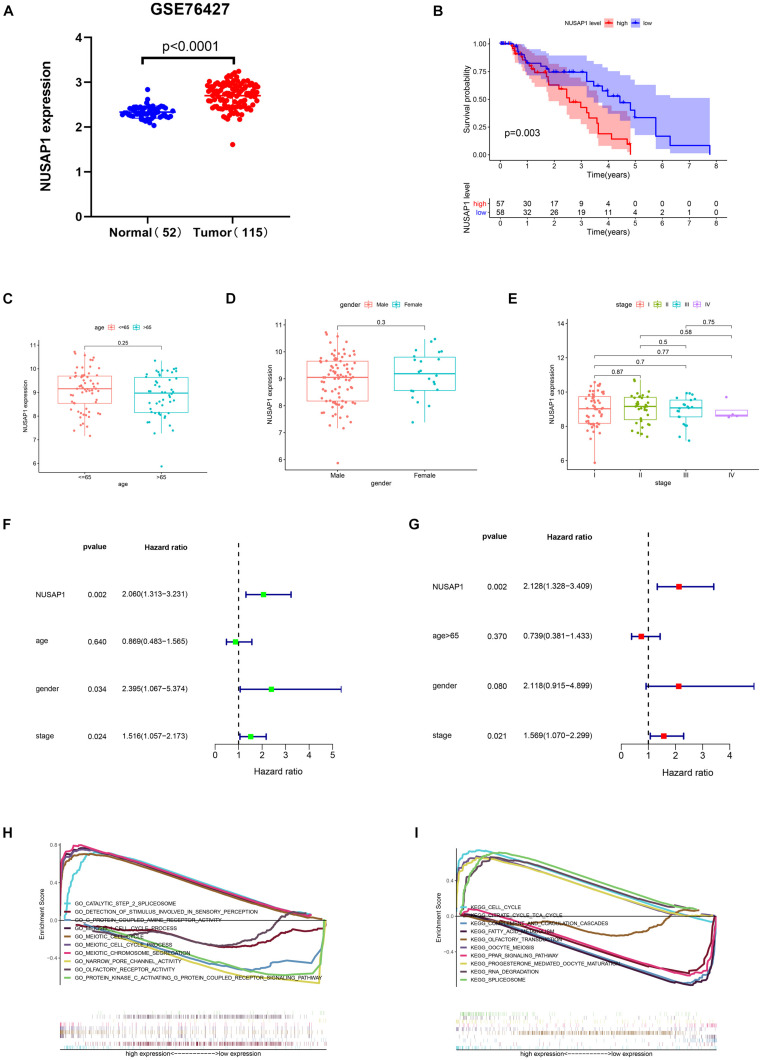
Dot plot, Kaplan–Meier curve, box plots, forest plots, and GSEA enrichment analysis. All pictures were made by HCC patients’ expression and clinical information in GSE76427 dataset. **(A)** Dot plot of NUSAP1 expression in normal samples and HCC samples. **(B)** Kaplan–Meier curve of OS analyzed between high NUSAP1 expression group and low expression group. **(C)** Bar graph of NUSAP1 expression in different age groups. **(D)** Bar graph of NUSAP1 expression in female and male. **(E)** Bar graph of NUSAP1 expression in different stage groups. **(F)** Univariate regression analysis. **(G)** Multivariate regression analysis. **(H)** GSEA enrichment analysis, which was based on GO gene sets. **(I)** GSEA enrichment analysis, which was based on KEGG gene sets.

Firstly, we generated a dot plot (Figure 3A) according to expression level of NUSAP1 in 52 normal samples and 115 HCC samples by GraphPad Prism; the result showed that expression level of NUSAP1 in tumor samples was significantly higher than that in normal samples (*p* < 0.0001). Next, we divided 155 HCC samples into high expression group and low expression group according to median expression level of NUSAP1 and draw Kaplan–Meier curve of OS (Figure 3B). The Kaplan–Meier curve showed that OS of the high expression group was significantly lower than low expression group (*p* = 0.003), which indicated that HCC patients with higher expression level of NUSAP1 had a worse prognosis. Subsequently, we continued to explore the relationships between 155 HCC patients’ age (Figure 3C), gender (Figure 3D), stage (Figure 3E), and expression level of NUSAP1. The results showed that there was no significant relationship between expression level of NUSAP1 and age, gender, or stage. Besides, in order to further explore whether expression level of NUSAP1 can be used as an independent prognostic factor, we conducted univariate (Figure 3F) and multivariate regression analysis (Figure 3G) using clinical information of 155 HCC patients. The results showed NUSAP1 was statistically significant in univariate regression analysis (*p* = 0.002, HR = 2.060) and multivariate regression analysis (*p* = 0.002, HR = 2.128); it verified that NUSAP1 was an independent prognostic factor of HCC. At last, to further explore underlying mechanism of NUSAP1 promoting HCC progress, we divided the HCC patients into high expression group and low expression group according to expression level of NUSAP1 and performed GSEA enrichment analyses based on GO and KEGG gene sets, respectively. The top 5 upregulated gene enrichment results and top 5 downregulated gene enrichment results based on GO gene sets were shown in Figure 3H; another top 5 upregulated gene enrichment results and top 5 downregulated gene enrichment results based on KEGG gene sets were exhibited in Figure 3I. By analyzing two results of GSEA, we found that NUSAP1 may promote HCC progression by regulating cell cycle.

### Survival Analysis, Clinical Correlation Analysis, Independent Prognostic Analysis, and GSEA Enrichment Analysis of NUSAP1 by HCC Patients in ICGC Database

**FIGURE 4:**
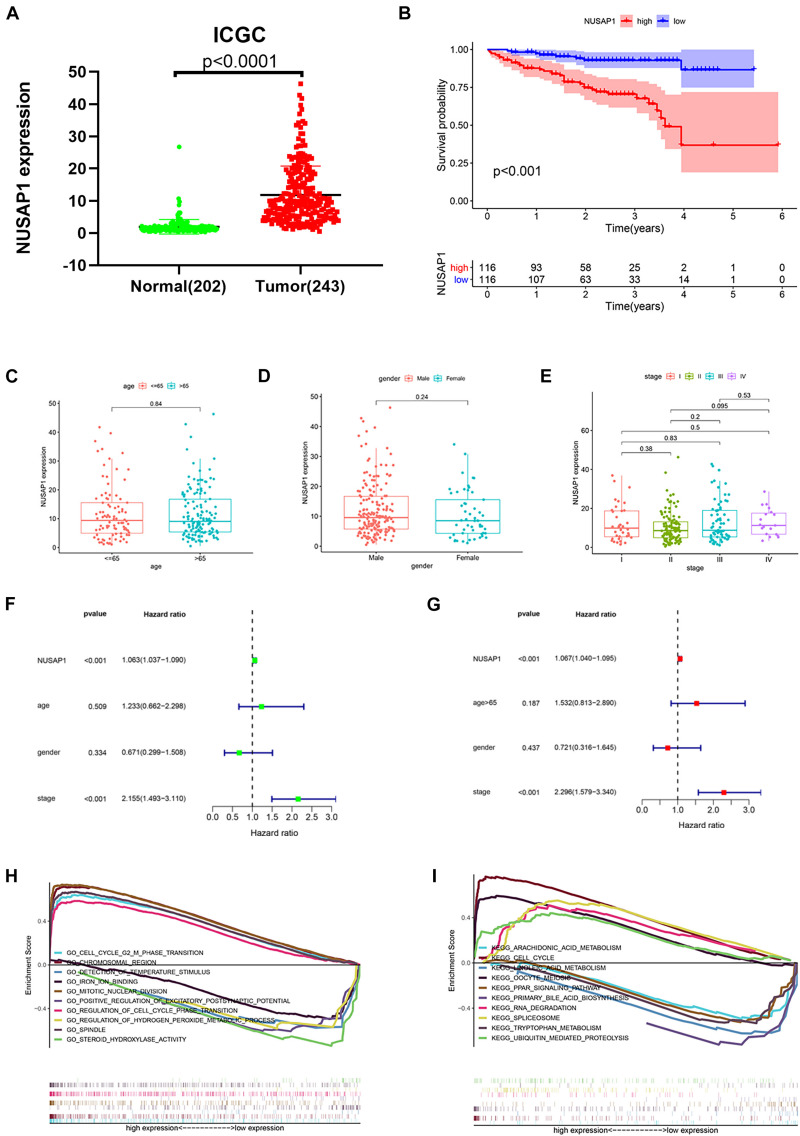
Dot plot, Kaplan–Meier curve, box plots, forest plots, and GSEA enrichment analysis. All pictures were made by HCC patients’ expression and clinical information in ICGC database. **(A)** Dot plot of NUSAP1 expression in normal samples and HCC samples. **(B)** Kaplan–Meier curve of OS analyzed between high NUSAP1 expression group and low expression group. **(C)** Bar graph of NUSAP1 expression in different age groups. **(D)** Bar graph of NUSAP1 expression in female and male. **(E)** Bar graph of NUSAP1 expression in different stage groups. **(F)** Univariate regression analysis. **(G)** Multivariate regression analysis. **(H)** GSEA enrichment analysis, which was based on GO gene sets. **(I)** GSEA enrichment analysis, which was based on KEGG gene sets.

In order to test results that we generated by GSE76427 dataset, we downloaded 202 normal liver samples’ gene expression levels and 243 HCC samples’ gene expression levels and clinical information from ICGC database. Firstly, we drew a dot plot (Figure 4A) according to NUSAP1 expression level in 202 normal samples and 243 HCC samples using GraphPad Prism; the result showed expression level of NUSAP1 in tumor samples was significantly higher than that in normal samples (*p* < 0.0001), which was same as the result we got by GSE76427 dataset. Subsequently, we divided 243 HCC samples into high expression group and low expression group according to median expression of NUSAP1 and draw Kaplan–Meier curve of OS (Figure 4B). The Kaplan–Meier curve showed that OS of the high expression group was significantly lower than low expression group (*p* < 0.001), which further indicated that HCC patients with higher expression level of NUSAP1 had a worse prognosis. Next, we also explore the relationships between 243 HCC patients’ age (Figure 4C), gender (Figure 4D), or stage (Figure 4E) and expression level of NUSAP1; the results showed that there was no significant relationship between expression level of NUSAP1 and age, gender, or stage. We continued to conduct univariate (Figure 4F) and multivariate regression analysis (Figure 4G) using clinical information of 243 HCC patients; the results showed NUSAP1 was statistically significant in univariate regression analysis (*p* < 0.001, HR = 1.063) and multivariate regression analysis (*p* < 0.001, HR = 1.067), which verified that NUSAP1 was an independent prognostic factor of HCC. Lastly, to further explore underlying mechanism of NUSAP1 promoting HCC progress, we divided 243 HCC patients into high expression group and low expression group according to expression level of NUSAP1 and performed GSEA enrichment analyses based on GO and KEGG gene sets respectively. The top 5 upregulated gene enrichment results and top 5 downregulated gene enrichment results based on GO gene sets were shown in Figure 4H; another top 5 upregulated gene enrichment results and top 5 downregulated gene enrichment results based on KEGG gene sets were exhibited in Figure 4I. By analyzing two results, we once again proved that NUSAP1 promoted tumor progression by regulating cell cycle.

### Survival Analysis, Clinical Correlation Analysis, Independent Prognostic Analysis, and GSEA Enrichment Analysis of NUSAP1 by HCC Patients in TCGA Database

**FIGURE 5:**
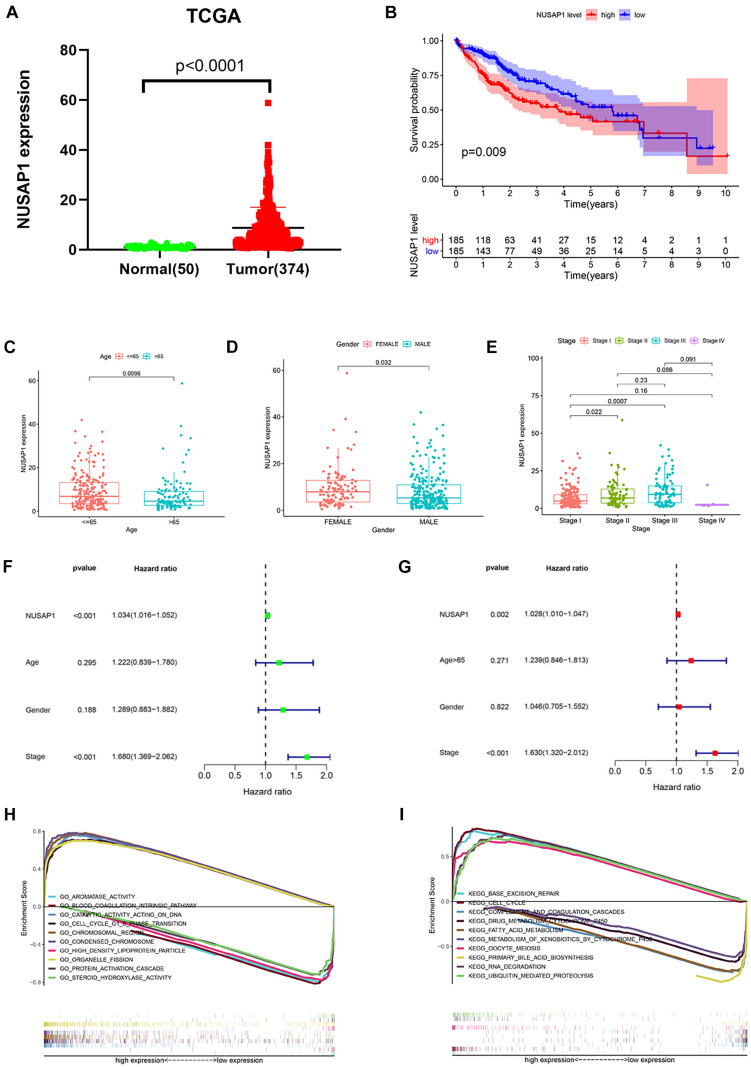
Dot plot, Kaplan–Meier curve, box plots, forest plots, and GSEA enrichment analysis. All pictures were made by HCC patients’ expression and clinical information in TCGA database. **(A)** Dot plot of NUSAP1 expression in normal samples and HCC samples. **(B)** Kaplan–Meier curve of OS analyzed between high NUSAP1 expression group and low expression group. **(C)** Bar graph of NUSAP1 expression in different age groups. **(D)** Bar graph of NUSAP1 expression in female and male. **(E)** Bar graph of NUSAP1 expression in different stage groups. **(F)** Univariate regression analysis. **(G)** Multivariate regression analysis. **(H)** GSEA enrichment analysis, which was based on GO gene sets. **(I)** GSEA enrichment analysis, which was based on KEGG gene sets.

Besides, we also we downloaded 50 normal liver samples’ gene expression levels and 374 HCC samples’ gene expression levels and clinical information from TCGA database to test our results, which were generated by GSE76427 dataset. The dot plot (Figure 5A) of NUSAP1 expression level in 50 normal samples and 374 HCC samples showed expression level of NUSAP1 in tumor samples was significantly higher than that in normal samples (*p* < 0.0001), which was same as the result we got by GSE76427 dataset. The Kaplan–Meier curve of NUSAP1 (Figure 5B) also showed that OS of the high expression group was significantly lower than low expression group (*p* = 0.009), which further indicated that HCC patients with higher expression level of NUSAP1 had a worse prognosis. Next, we also explore the relationships between 243 HCC patients’ age (Figure 5C), gender (Figure 5D), or stage (Figure 5E) and expression level of NUSAP1; the results showed that NUSAP1 expression level was different between patients older than 65 years and patients younger than 65 years (*p* = 0.0098), and it was also different between females and males (*p* = 0.032), between stage I and stage II (*p* = 0.022), and stage I and stage III (*p* = 0.0007). We continued to conduct univariate (Figure 5F) and multivariate regression analysis (Figure 5G) using clinical information of 374 HCC patients; the results showed NUSAP1 was statistically significant in univariate regression analysis (*p* < 0.001, HR = 1.034) and multivariate regression analysis (*p* < 0.002, HR = 1.028), which once again verified that NUSAP1 was an independent prognostic factor of HCC. Finally, to further explore underlying mechanism of NUSAP1 promoting HCC progress, we divided 374 HCC patients into high expression group and low expression group according to expression level of NUSAP1 and performed GSEA enrichment analyses based on GO and KEGG gene sets, respectively. The top 5 upregulated gene enrichment results and top 5 downregulated gene enrichment results based on GO gene sets were shown in Figure 5H; another top 5 upregulated gene enrichment results and top 5 downregulated gene enrichment results based on KEGG gene sets were exhibited in Figure 5I. By analyzing two results, we once again proved that NUSAP1 promoted tumor progression by regulating cell cycle and it was associated with G1/S phase transition.

### Survival Analysis, Clinical Correlation Analysis, Independent Prognostic Analysis, and GSEA Enrichment Analysis of NUSAP1 by Uniting HCC Patients in GSE76427 Dataset, ICGC Database, and TCGA Database

**FIGURE 6:**
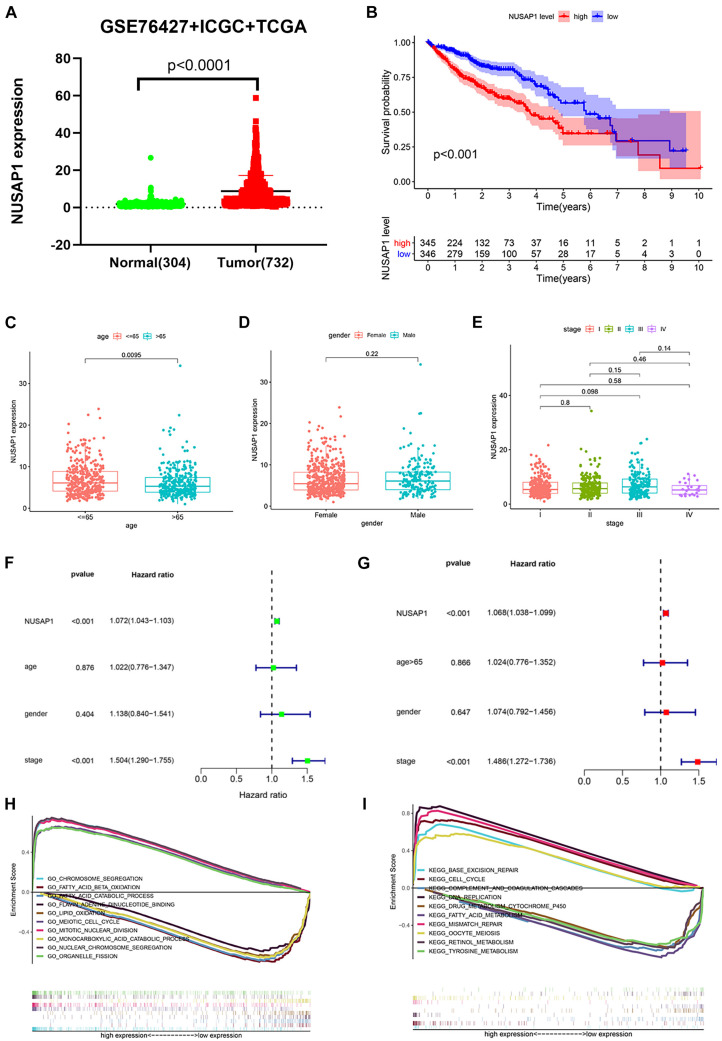
Dot plot, Kaplan–Meier curve, box plots, forest plots, and GSEA enrichment analysis. All pictures were made by HCC patients’ expression and clinical information in GSE76427 dataset, ICGC database, and TCGA database. **(A)** Dot plot of NUSAP1 expression in normal samples and HCC samples. **(B)** Kaplan–Meier curve of OS analyzed between high NUSAP1 expression group and low expression group. **(C)** Bar graph of NUSAP1 expression in different age groups. **(D)** Bar graph of NUSAP1 expression in female and male. **(E)** Bar graph of NUSAP1 expression in different stage groups. **(F)** Univariate regression analysis. **(G)** Multivariate regression analysis. **(H)** GSEA enrichment analysis, which was based on GO gene sets. **(I)** GSEA enrichment analysis, which was based on KEGG gene sets.

In order to make our results more scientific and convincing, we merged all normal samples’ gene expression level and HCC samples’ gene expression level clinical information from GSE76427 dataset, ICGC database, and TCGA database, which contained 304 normal samples and 732 HCC samples. Subsequently, we used the merged dataset to test all the results we generated before. The dot plot (Figure 6A) of NUSAP1 expression level in 304 normal samples and 732 HCC samples showed expression level of NUSAP1 in tumor samples was significantly higher than that in normal samples (*p* < 0.0001). The Kaplan–Meier curve of NUSAP1 (Figure 6B) showed that OS of the high expression group was significantly lower than low expression group (*p* < 0.001), which indicated that HCC patients with higher expression level of NUSAP1 had a worse prognosis. Next, we continued to explore the relationships between 732 HCC patients’ age (Figure 6C), gender (Figure 6D), or stage (Figure 6E) and expression level of NUSAP1; the results showed that NUSAP1 expression level was different between patients older than 65 years and patients younger than 65 years (*p* = 0.0095). There was no significant relationship between expression level of NUSAP1, gender, and stage. We continued to conduct univariate (Figure 6F) and multivariate regression analysis (Figure 6G) using clinical information of 732 HCC patients; the results showed NUSAP1 was statistically significant in univariate regression analysis (*p* < 0.001, HR = 1.072) and multivariate regression analysis (*p* < 0.002, HR = 1.068), which demonstrated that NUSAP1 was an independent prognostic factor of HCC again. Finally, to further explore underlying mechanism of NUSAP1 promoting HCC progress, we divided 732 HCC patients into high expression group and low expression group according to expression level of NUSAP1 and performed GSEA enrichment analyses based on GO and KEGG gene sets, respectively. The top 5 upregulated gene enrichment results and top 5 downregulated gene enrichment results based on GO gene sets were shown in Figure 6H; another top 5 upregulated gene enrichment results and top 5 downregulated gene enrichment results based on KEGG gene sets were exhibited in Figure 6I. By analyzing two results, we once again demonstrated that NUSAP1 promoted tumor progression by regulating cell cycle.

### Screening Co-expression Genes of NUSAP1 in HCC and Performed GO and KEGG Enrichment Analysis

**FIGURE 7:**
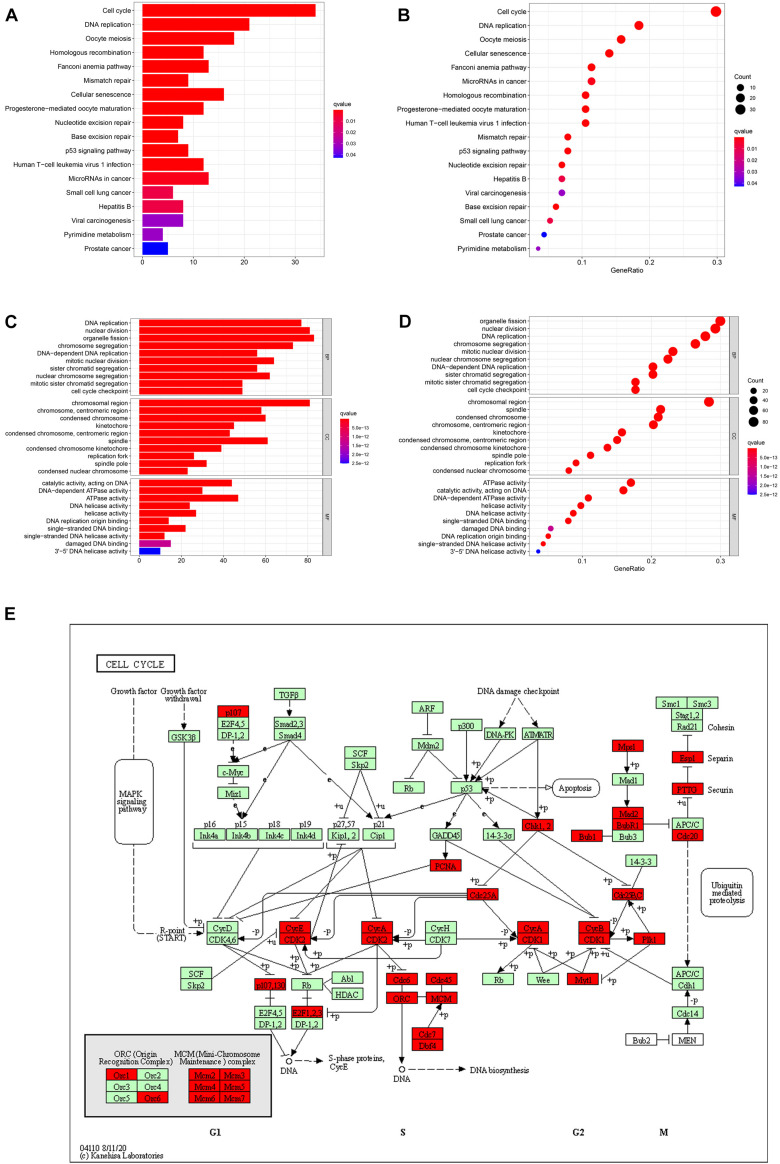
Bar graphs, bubble diagrams, and cell cycle pathway map. **(A)** Bar graph of KEGG enrichment analysis. **(B)** Bubble diagram of KEGG enrichment analysis. **(C)** Bar graph of GO enrichment analysis. **(D)** Bubble diagram of GO enrichment analysis. **(E)** Cell cycle pathway map; the co-expression genes of NUSAP1 were mainly existed in S phase and G2/M phase of the cell cycle.

By analyzing three datasets before, we confirmed that NUSAP1 was an independent prognostic factor of HCC and closely associated with HCC patients’ OS; its underlying mechanism was related to cell cycle. Hence, to further explore and verify its underlying mechanism, we used cBioProtal online data analysis tool to screen all co-expression genes of NUSAP1 (Pearson scores > 0.3, Spearman scores > 0.3). Subsequently, we used top 300 co-expression genes to accomplish KEGG and GO enrichment analysis by DAVID online data analysis tool and visualized KEGG result (Figures 7A,B) and GO result (Figures 7C,D) by bar plots and bubble plots using R studio. The results also indicated that expression of NUSAP1 mainly promoted HCC progress by regulating cell cycle. Subsequently, we continued to draw KEGG pathway map of cell cycle (Figure 7E) using co-expression genes, which were enriched in cell cycle pathway by KEGG online tool. The map depicted that most of co-expression genes of NUSAP1 were located in S phase and G2/M phase of the cell cycle, and they could regulate the genes in G1 phase. Herein, we speculated that NUSAP1 may regulate progression of HCC mainly by promoting the transition from the G1 phase to S phase transition. We thought the conclusion was of great clinical significance; if there was a targeted therapy drug that could inhibit the function of NUSAP1 to promote G1 to S phase transition, it was possible to inhibit the progression of HCC.

### NUSAP1 Was Overexpressed in Huh7 and HepG2 Cell Lines and Related to Cell Cycle

**FIGURE 8:**
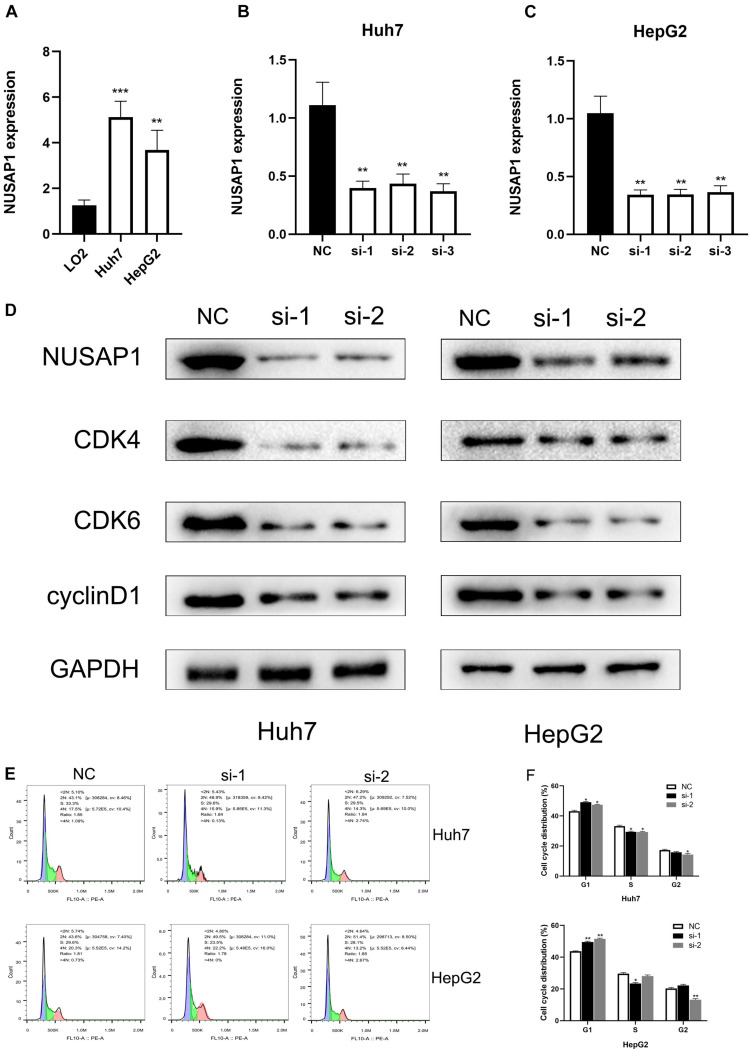
qRT-PCR, Western blot, and flow cytometry. **(A)** Bar graph of qRT-PCR, which was used to examine NUSAP1 expression level in normal human hepatic cell line LO2, HCC cell line Huh7, and HCC cell line HepG2. **(B)** Bar graph of qRT-PCR, which was used to examine NUSAP1 expression level among NC control group, si-1 treatment group, si-2 treatment group, and si-3 treatment group in Huh7 cell line. **(C)** Bar graph of qRT-PCR, which was used to examine NUSAP1 expression level among NC control group, si-1 treatment group, si-2 treatment group, and si-3 treatment group in HepG2 cell line. **(D)** Western blot was performed to measure the expression of CDK4, CDK6, and cyclinD1, which were crucial protein for regulating G1 to S phase transition. **(E)** Flow cytometric analysis of cell cycle distribution in HepG2 and Huh7 cell lines treated with siNUSAP1. **(F)** By comparing with NC group, si-1 and si-2 groups increased cell cycle arrest at the G1 phase. *P < 0.05; **P < 0.01; ***P < 0.001.

In order to further prove conclusions that we obtained by multi-database analysis, we continued to carry out relevant cell lines experiments. Firstly, to improve NUSAP1 expression level whether it had significant difference between normal liver cells and HCC cells, we examined NUSAP1 expression level in normal human hepatic cell line LO2, HCC cell line Huh7, and HCC cell line HepG2 (Figure 8A) by qRT-PCR. The result showed that the expression level of NuSAP1 was observably higher in Huh7 and HepG2 cell lines compared with LO2 cell line, which was consistent with the outcomes we got from multi-database before. Subsequently, we continued to silence NUSAP1 in Huh7 and HepG2 cell lines by siRNA transfection, respectively, and divided each cell line into NC (Negative Control) control group, si-1 treatment group, si-2 treatment group, and si-3 treatment group. We examined NUSAP1 expression level among NC control group, si-1 treatment group, si-2 treatment group, and si-3 treatment group in Huh7 cell line (Figure 8B) by RT-qPCR analysis; the result revealed that NUSAP1 expression level in NC group was significantly higher than si-1, si-2, and si-3 groups. We continued to examine NUSAP1 expression level among NC control group, si-1 treatment group, si-2 treatment group, and si-3 treatment group in HepG2 cell line (Figure 8C) by RT-qPCR analysis; the result also revealed that NUSAP1 expression level in NC group was significantly higher than si-1, si-2, and si-3 group. Hence, Western blot was performed to measure the expression of CDK4, CDK6, and cyclinD1, which were crucial protein for regulating G1 to S phase transition (Figure 8D). The results revealed the expression of NUSAP1 was positively associated with CDK4, CDK6, and cyclinD1, indicating that NUSAP1 promoted HCC progression by regulating G1 to S phase transition.

To further verify the effect of NUSAP1 on cell cycle transition, we did flow cytometry using HepG2 and Huh7 cell lines. Comparing with the NC group, si-1 and si-2 groups significantly increased the proportion of G1-phase cells in both HepG2 and Huh7 cells (Figures 8E,F). The result indicated that NUSAP1 silencing may decrease cell cycle arrest at G1 phase; it validated our precious conclusion that NUSAP1 promoted HCC progression by regulating G1 to S phase transition again.

### Analyzing Correlations Between Nusap1 and Immune Cells by HCC Patients in GSE76427 Dataset

**FIGURE 9:**
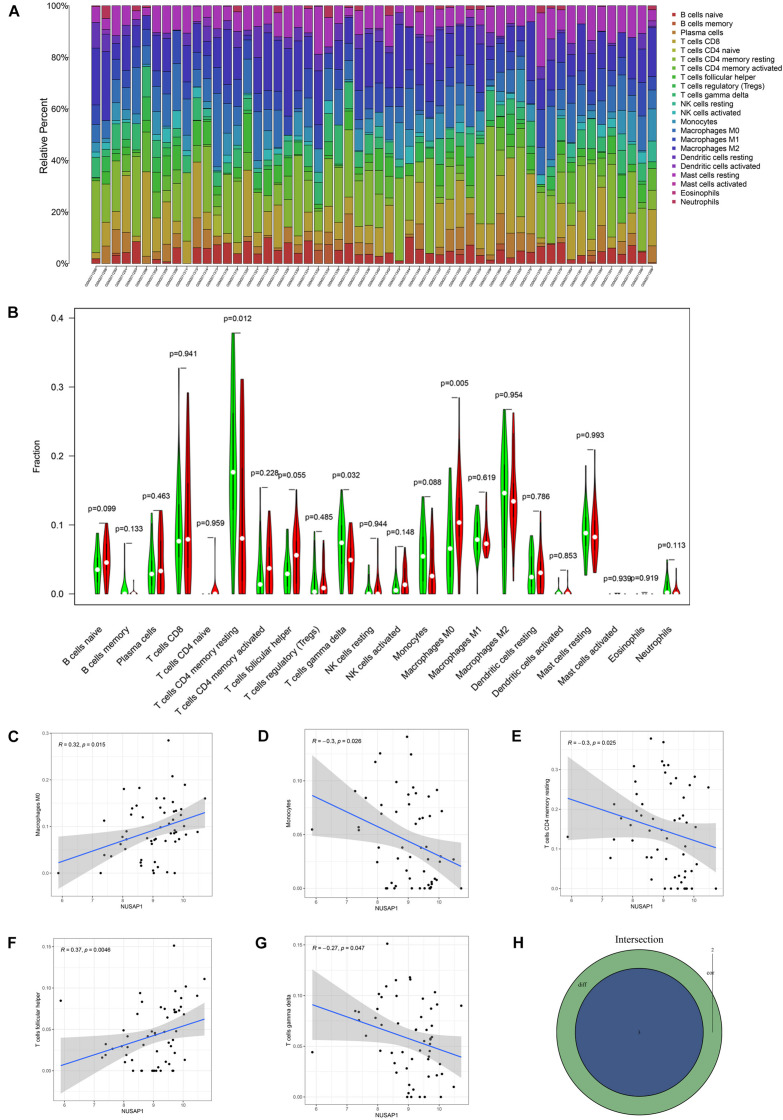
Bar plot, violin plot, correlation diagrams, and Venn diagram. All pictures were drawn by HCC patients’ information in GSE76427 dataset. **(A)** Bar graph, which contained content of 22 immune cells in each sample. **(B)** Violin plot, which proved 22 immune cells whether they exhibited difference between high expression group and low expression group. **(C)** The correlation diagram between NUSAP1 and macrophages M0. **(D)** The correlation diagram between NUSAP1 and monocytes. **(E)** The correlation diagram between NUSAP1 and T cells CD4 memory resting. **(F)** The correlation diagram between NUSAP1 and T cells follicular helper. **(G)** The correlation diagram between NUSAP1 and T cells gamma delta. **(H)** Venn diagram, which was used to visualize the immune cells that were both meaningful in violin plot and correlation diagrams.

Subsequently, we further explored correlations between NUSAP1 and immune cells in HCC patients. Firstly, we draw a bar plot that contained content of 22 immune cells (B cells naive, B cells memory, plasma cells, T cells CD8, T cells CD4 naive, T cells CD4 memory resting, T cells CD4 memory activated, T cells follicular helper, T cells regulatory (Tregs), T cells gamma delta, NK cells resting, NK cells activated, monocytes, macrophages M0, macrophages M1, macrophages M2, dendritic cells resting, dendritic cells activated, mast cells resting, mast cells activated, eosinophils, neutrophils) in each sample by R studio (Figure 9A). Next, we divided 155 HCC samples into high expression group and low expression group according to median expression level of NUSAP1 and drew violin plot, which was used to analyze 22 immune cells whether they exhibited difference between high expression group and low expression group (Figure 9B). The result revealed that T cells CD4 memory resting (*p* = 0.012), T cells gamma delta (*p* = 0.032), and macrophages M0 (*p* = 0.005) were significantly different between high and low expression group. Besides, we also generated correlation diagrams between NUSAP1 and 22 immune cells and the meaningful diagrams were shown in figure, which concluded macrophages M0 (Figure 9C), monocytes (Figure 9D), T cells CD4 memory resting (Figure 9E), T cells follicular helper (Figure 9F), and T cells gamma delta (Figure 9G). At last, we obtained immune cells that were both meaningful in violin plot and correlation diagrams and visualized by Venn diagram (Figure 9H). The overlapping immune cells contained T cells CD4 memory resting, T cells gamma delta, and macrophages M0.

### Analyzing Correlations Between NUSAP1 and Immune Cells by HCC Patients in ICGC Database

**FIGURE 10:**
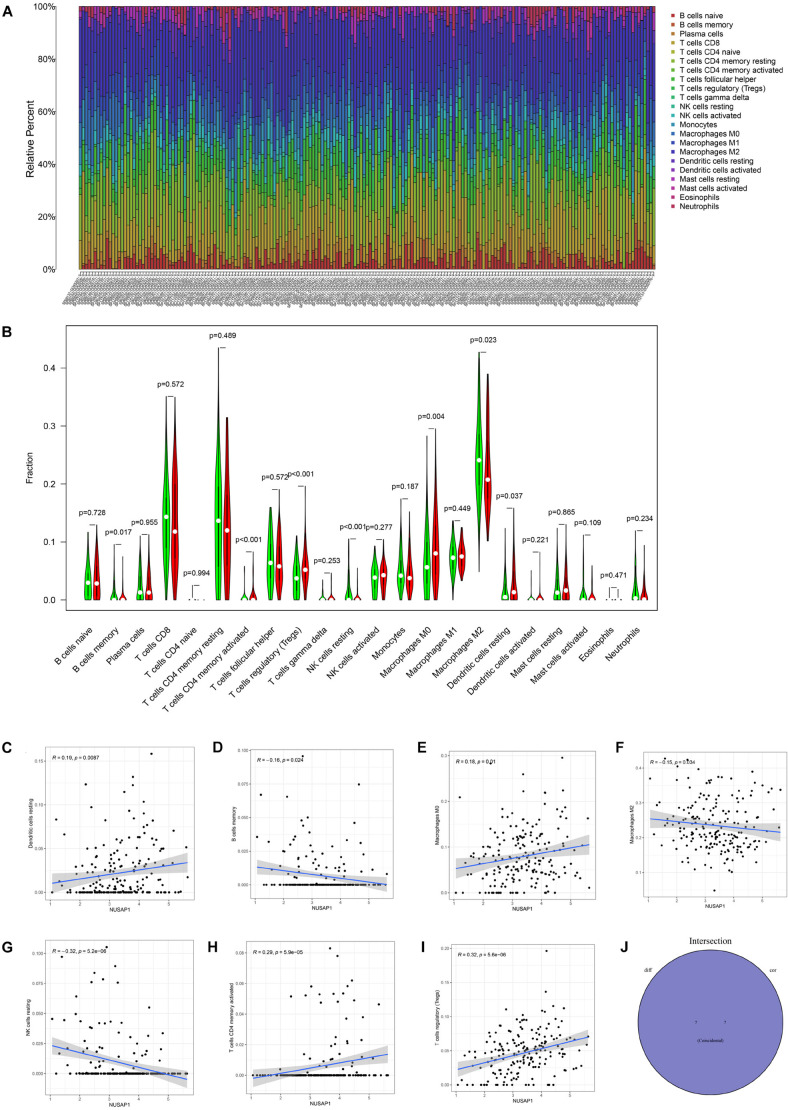
Bar plot, violin plot, correlation diagrams, and Venn diagram. All pictures were drawn by HCC patients’ information in ICGC database. **(A)** Bar graph, which contained content of 22 immune cells in each sample. **(B)** Violin plot, which proved 22 immune cells whether they exhibited difference between high expression group and low expression group. **(C)** The correlation diagram between NUSAP1 and dendritic cells resting. **(D)** The correlation diagram between NUSAP1 and B cells memory. **(E)** The correlation diagram between NUSAP1 and macrophages M0. **(F)** The correlation diagram between NUSAP1 and macrophages M2. **(G)** The correlation diagram between NUSAP1 and NK cells resting. **(H)** The correlation diagram between NUSAP1 and T cells CD4 memory activated. **(I)** The correlation diagram between NUSAP1 and T cells regulatory (Tregs). **(J)** Venn diagram, which was used to visualize the immune cells that were both meaningful in violin plot and correlation diagrams.

We continued to analyze correlations between NUSAP1 and immune by HCC patients in ICGC database. Similarly, we draw a bar plot which contained content of 22 immune cells in each sample by R studio (Figure 10A). Next, we divided 243 HCC samples into high expression group and low expression group according to median expression level of NUSAP1 and drew violin plot, which was used to analyze 22 immune cells whether they exhibited difference between high expression group and low expression group (Figure 10B). The result revealed that B cells memory (*p* = 0.017), T cells CD4 memory activated (*p* = 0.037), T cells regulatory (Tregs) (*p* = 0.037), NK cells resting (*p* < 0.001), macrophages M0 (*p* = 0.004), macrophages M2 (*p* = 0.023), and dendritic cells resting (*p* = 0.037) were significantly different between high and low expression group. Besides, we also generated correlation diagrams between NUSAP1 and 22 immune cells and the meaningful diagrams were shown in figure, which concluded dendritic cells resting (Figure 10C), B cells memory (Figure 10D), macrophages M0 (Figure 10E), macrophages M2 (Figure 10F), NK cells resting (Figure 10G), T cells CD4 memory activated (Figure 10H), and T cells regulatory (Tregs) (Figure 10I). At last, we obtained immune cells that were both meaningful in violin plot and correlation diagrams and visualized by Venn diagram (Figure 10J). The overlapping immune cells contained B cells memory, T cells CD4 memory activated, T cells regulatory (Tregs), NK cells resting, macrophages M0, macrophages M2, and dendritic cells resting.

### Analyzing Correlations Between NUSAP1 and Immune Cells by HCC Patients in TCGA Database

**FIGURE 11:**
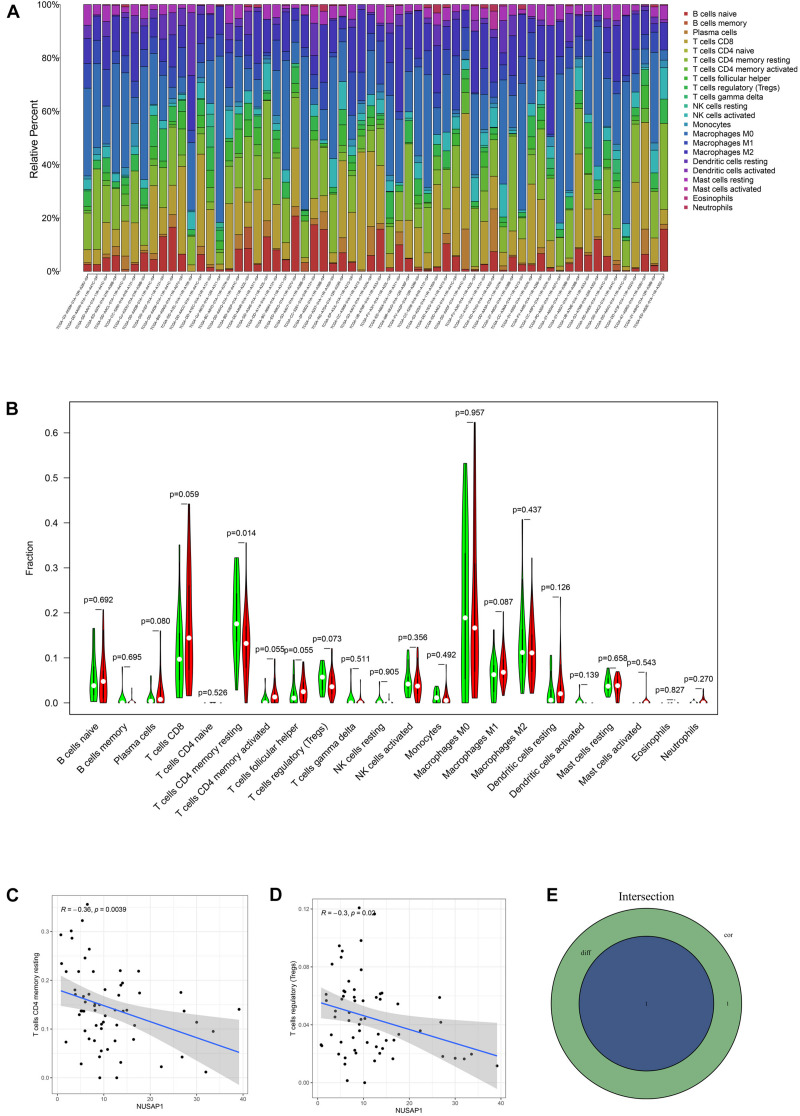
Bar plot, violin plot, correlation diagrams, and Venn diagram. All pictures were drawn by HCC patients’ information in TCGA database. **(A)** Bar graph, which contained content of 22 immune cells in each sample. **(B)** Violin plot, which proved 22 immune cells whether they exhibited difference between high expression group and low expression group. **(C)** The correlation diagram between NUSAP1 and T cells CD4 memory resting. **(D)** The correlation diagram between NUSAP1 and T cells regulatory (Tregs). **(E)** Venn diagram, which was used to visualize the immune cells that were both meaningful in violin plot and correlation diagrams.

To analyze correlations between NUSAP1 and immune by HCC patients in TCGA database, firstly, we draw a bar plot, which contained content of 22 immune cells in each sample by R studio (Figure 11A). Next, we divided 374 HCC samples into high expression group and low expression group according to median expression level of NUSAP1 and drew violin plot, which was used to analyze 22 immune cells whether they exhibited difference between high expression group and low expression group (Figure 11B). The result revealed that only T cells CD4 memory resting (*p* = 0.014) were significantly different between high and low expression group. Besides, we also generated correlation diagrams between NUSAP1 and 22 immune cells and the meaningful diagrams were shown in figure, which only concluded T cells CD4 memory resting (Figure 11C) and T cells regulatory (Tregs) (Figure 11D). At last, we obtained immune cells that were both meaningful in violin plot and correlation diagrams and visualized by Venn diagram (Figure 11E). The overlapping immune cells only contained T cells CD4 memory resting.

### Analyzing Correlations Between NUSAP1 and Immune Cells by Uniting HCC Patients in GSE76427 Dataset, ICGC Database, and TCGA Database

**FIGURE 12:**
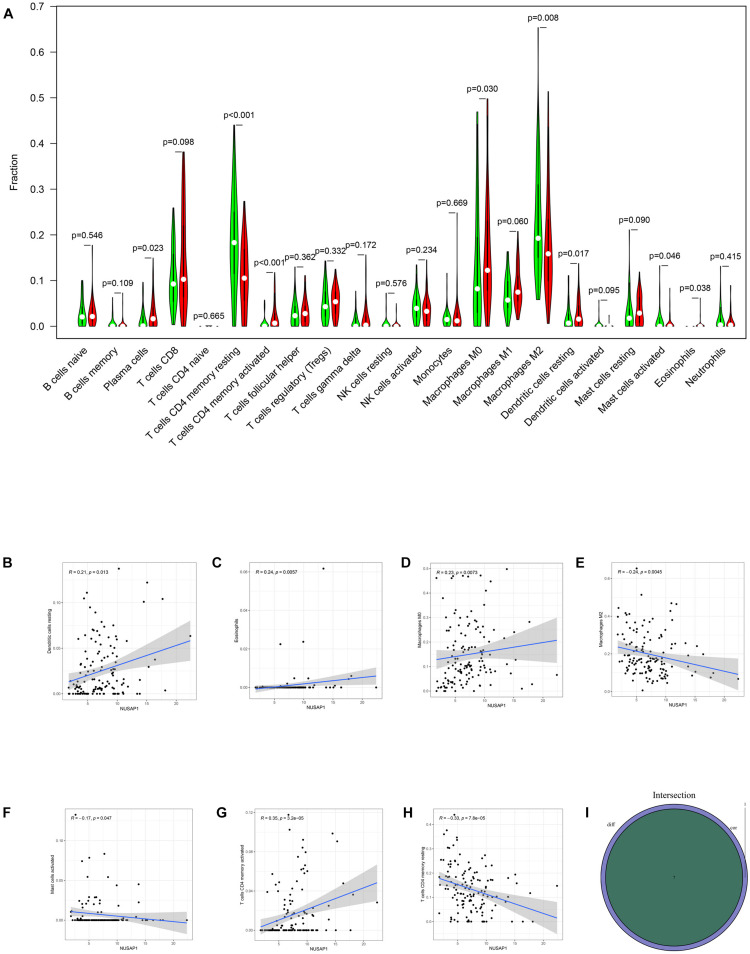
Bar plot, violin plot, correlation diagrams, and Venn diagram. All pictures were drawn by HCC patients’ information in GSE76427 dataset, ICGC database, and TCGA database. **(A)** Violin plot, which proved 22 immune cells whether they exhibited difference between high expression group and low expression group. **(B)** The correlation diagram between NUSAP1 and dendritic cells resting. **(C)** The correlation diagram between NUSAP1 and eosinophils. **(D)** The correlation diagram between NUSAP1 and macrophages M0. **(E)** The correlation diagram between NUSAP1 and macrophages M2. **(F)** The correlation diagram between NUSAP1 and mast cells activated. **(G)** T cells CD4 memory activated **(H)** T cells CD4 memory resting**. (I)** Venn diagram, which was used to visualize the immune cells that were both meaningful in violin plot and correlation diagrams.

Firstly, we divided 732 HCC samples into high expression group and low expression group according to median expression level of NUSAP1 and drew violin plot, which was used to analyze 22 immune cells whether they exhibited difference between high expression group and low expression group (Figure 12A). The result revealed that plasma cells (*p* = 0.023), T cells CD4 memory resting (*p* < 0.001), T cells CD4 memory activated (*p* < 0.001), macrophages M0 (*p* = 0.030), macrophages M2 (*p* = 0.008), dendritic cells resting (*p* = 0.017), mast cells activated (*p* = 0.046), and eosinophils (*p* = 0.038) were significantly different between high and low expression group. We also generated correlation diagrams between NUSAP1 and 22 immune cells, and the meaningful diagrams were shown in figure, which only concluded dendritic cells resting (Figure 12B), eosinophils (Figure 12C), macrophages M0 (Figure 12D), macrophages M2 (Figure 12E), mast cells activated (Figure 12F), T cells CD4 memory activated (Figure 12G), and T cells CD4 memory resting (Figure 12H). At last, we obtained immune cells that were both meaningful in violin plot and correlation diagrams and visualized by Venn diagram (Figure 12I). The overlapping immune cells included dendritic cells resting, eosinophils, macrophages M0, macrophages M2, mast cells activated, T cells CD4 memory activated, and T cells CD4 memory resting. Therefore, through the combination of multiple databases analysis, we speculated that NUSAP1 also could promote HCC progress by influencing T cells CD4 memory resting and macrophages M0 through some underlying mechanism.

### Analyzing Correlations Between NUSAP1 and Four Immune Checkpoint Molecules (CTLA4, PD1, PD-L1, and PD-L2)

**FIGURE 13:**
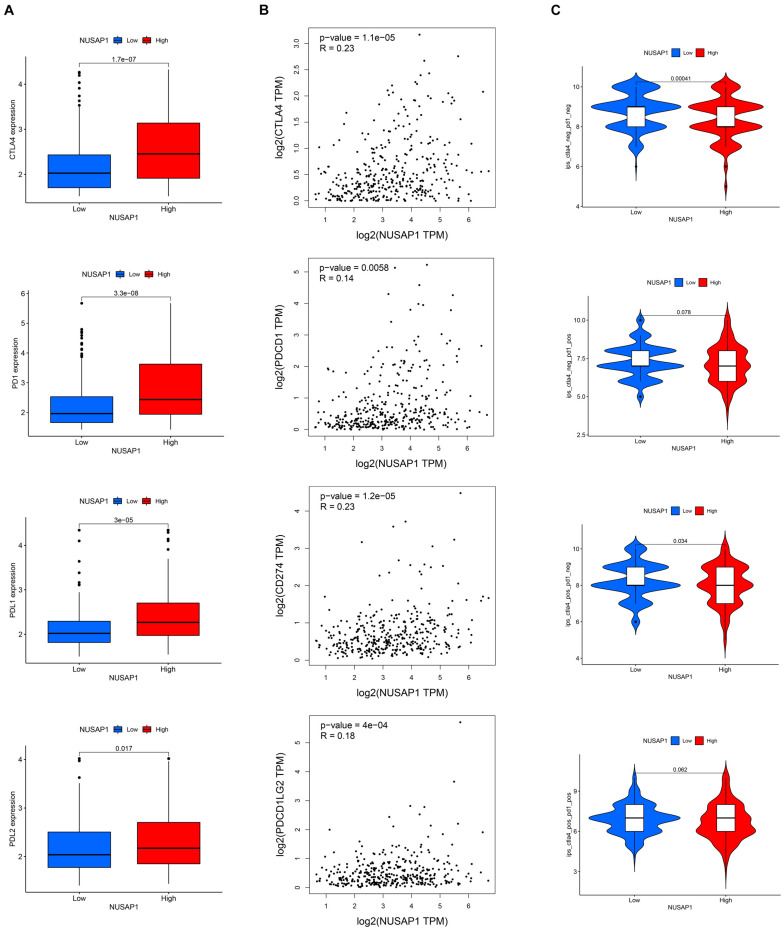
The estimation of immunotherapy response between high and low expression group of NUSAP1. **(A)** The expression level of the four immune checkpoint molecules (CTLA4, PD1, PD-L1, and PD-L2) in high and low expression groups. **(B)** The Pearson correlation coefficient between NUSAP1 and the four immune checkpoint molecules. **(C)** The IPS in high and low expression groups.

Besides, we also continued to explore correlations between NUSAP1 and four immune checkpoint molecules. Firstly, we analyzed the expression level of the four immune checkpoint molecules between high and low expression group of NUSAP1 in TCGA database. The results showed that high expression group presented with a higher level of immune checkpoint molecules (Figure 13A). Then, we found that there were positive correlations between NUSAP1 and the four immune checkpoint molecules (Figure 13B), which were analyzed by Gene Expression Profiling Interactive Analysis (GEPIA)^10^ basing on TCGA database. Lastly, the immunogenicity of the two groups was assessed by IPS analysis. The results showed that ips_ctla4_neg_pd1_neg and ips_ctla4_pos_pd1_neg scores were higher in the low expression group (Figure 13C). Thus, we concluded that HCC patients in the low expression group of NUSAP1 might present with a better response for CTLA4 immunotherapy.

## Discussion

HCC is the sixth most common and the fourth deadliest malignant tumors globally; it has a serious impact on human health. Herein, to study the relationships between key gene and occurrence, development or prognosis of HCC is absolutely a necessary affair; it may guide us to find a new molecular marker in HCC, the underlying molecular mechanisms of HCC, and even a new treatment option at the molecular and genetic level of HCC.

Herein, four gene chip datasets of HCC (GSE62232, GSE102079, GSE112790, and GSE121248) from GEO database were screened for bioinformatic analysis, and 86 common DEGs in the four datasets were identified. We used the 86 common DEGs to make a PPI network by String online tool. Then, the top 10 hub genes in PPI network were screened by cytoHubba plug-in in Cytoscope using 12 different calculating methods; thus, we gain 12 different outcomes of the top hub genes. By analyzing the 12 results, we screened our key gene NUSAP1.

Nucleolar and spindle-associated protein 1 (NUSAP1) is a nucleolar-spindle-associated protein that plays a role in spindle microtubule organization, which belongs to the NUSP1 family (Raemaekers et al., 2003; Petry, 2016). Previous studies had demonstrated that the overexpression of NUSAP1 was observed in many human neoplasms including colon cancer (Han et al., 2018; Liu et al., 2018), astrocytoma (Wu et al., 2017), glioblastoma multiforme (Qian et al., 2018), renal cell carcinoma (Fang et al., 2016), prostate cancer (Gulzar et al., 2013; Gordon et al., 2015, 2017), oral squamous cell carcinoma (Okamoto et al., 2015), breast cancer (Kretschmer et al., 2011; Chen et al., 2015), cervical carcinoma (Li et al., 2019), and esophageal squamous cell carcinoma (Guan et al., 2019). Besides, many studies also showed that overexpression of NUSAP1 was associated with poor survival of colon cancer (Liu et al., 2018), astrocytoma (Wu et al., 2017), glioblastoma multiforme (Qian et al., 2018), renal cell carcinoma (Fang et al., 2016), prostate cancer (Gordon et al., 2017), breast cancer (Chen et al., 2015), and esophageal squamous cell carcinoma (Guan et al., 2019). However, few studies had demonstrated the relationships between the expression of NUSAP1 and HCC. We found two studies that were related to NUSAP1 and HCC; one study demonstrated that NUSAP1 was a target of miRNA 193a-5p and microRNA 193a-5p can regulate levels of NUSAP1; HCC with low levels of miRNA 193a-5p could increase expression of NUSAP1, and the overexpression of NUSAP1 in HCC samples correlated with shorter survival times of patients (Roy et al., 2018). Another study was a transcriptome analysis; by analyzing microarray datasets incorporating cirrhosis and HCC subjects from GEO database, the author found that NUSAP1 was one of the top 5 significant genes that were associated with onset, progression, and prognosis of HCC and exhibited higher expression in HCC compared with normal livers (Shan et al., 2019). Herein, to further explore the relationships between the expression of NUSAP1 and HCC, we did the next series of study. Firstly, we did survival analysis, clinical correlation analysis, independent prognostic analysis, and GSEA enrichment analysis of NUSAP1 by HCC patients in GSE76427 dataset, ICGC database, and TCGA database; we verified that the higher expression of NUSAP1 was closely related with poorer prognosis; it was also an independent prognostic factor in HCC. Although we had demonstrated the relationships between expression of NUSAP1 and HCC patients’ survival, prognostic, the mechanism of NUSAP1 influencing HCC progress was not very clear until now. We found only one study reported that hepatitis B virus X protein can enhance hepatocarcinogenesis by depressing the targeting of NUSAP1 mRNA by miR-18b; the specific mechanism was the targeting of NUSAP1 mRNA by the tumor suppressor miR-18b, which was controlled by hepatitis B virus X modulated promoter methylation during the host–virus interaction, leading to hepatocarcinogenesis (Yang et al., 2019). Hence, in order to further explore the mechanism of NUSAP1 influencing HCC progress, we used HCC patients’ information from GSE76427 dataset, ICGC database, and TCGA database to accomplish GSEA enrichment analysis and found that NUSAP1 promoted tumor progression by regulating cell cycle. Besides, to further verify its underlying machine of cell cycle, we further did GO and KEGG enrichment analysis by co-expression genes of NUSAP1 through DAVID online tool, and we demonstrated again NUSAP1 was closely related to cell cycle. Subsequently, we continued to generate cell cycle KEGG pathway map using co-expression genes of NUSAP1 by KGGE online tool. The map depicted that most of co-expression genes of NUSAP1 were located in S phase and G2/M phase of the cell cycle, and they could regulate the genes in G1 phase. Herein, we speculated that NUSAP1 may regulate progression of HCC mainly by promoting the transition from the G1 phase to S phase transition. Besides, we also verified our conclusion through Western blotting and flow cytometry. We confirmed that our gene NUSAP1 was closely related to CDK4, CDK6, and cyclinD1, and NUSAP1 silencing decreased cell cycle arrest at G1 phase. Hence, this verified NUSAP1 was associated with G1 to S transition again.

Besides, we also analyzed correlations between NUSAP1 and immune cells by analyzing HCC patients in GSE76427 dataset, ICGC database, and TCGA database and we confirmed that NUSAP1 could also promote HCC progression by influencing T cells CD4 memory resting and macrophages M0 through some underlying mechanisms; however, the mechanisms were still waiting our exploration in the future. We also explored correlations between NUSAP1 and four immune modulators (CTLA4, PD1, PD-L1, and PD-L2), and we found that HCC patients in the low expression group of NUSAP1 might present with a better response for CTLA4 immunotherapy.

Through these analyses of multiple databases to NUSAP1, our study demonstrated the higher expression of NUSAP1 was associated with HCC patients’ shorter survival time and poorer prognosis. Besides, it was also an independent prognostic factor in HCC. Its underlying mechanism of promoting HCC progress was accelerating G1 to S phase transition. Besides, our study also speculated that the mechanism of NUSAP1 might also be associated with related immune mechanisms. However, it was regrettable that our study was almost a bioinformatics analysis. There were relatively few experiments *in vitro* to verify our results, and there was also a lack of further studies on its mechanism. Hence, we hope that more scholars will carry out further studies on its mechanism in the future.

## Conclusion

By integrating and analyzing four microarray datasets, we screened one key gene (NUSAP1), which was closely associated with survival and prognosis of HCC. Meanwhile, we further studied the mechanism of NUSAP1 in HCC by multi-database and found NUSAP1 played a significant role in regulating HCC progression by promoting the transition from the G1 phase to the S phase in cell cycle. Our results were of great clinical significance; it might provide some new ideas about early targeted therapy and prognostic judgment of HCC. Besides, NUSAP1 could also promote HCC progression by influencing T cells CD4 memory resting and macrophages M0 through some underlying mechanisms; however, the mechanisms were still waiting our exploration in the future.

## Data Availability Statement

The datasets presented in this study can be found in online repositories. The names of the repository/repositories and accession number(s) can be found in the article/supplementary material.

## Author Contributions

WZ, JX, and JJ designed the study. WZ and ZC collected the literature. WZ performed statistical analyses and wrote the manuscript. WZ, JX, ZC, and JJ analyzed the data. All authors read and approved the final manuscript.

## Conflict of Interest

The authors declare that the research was conducted in the absence of any commercial or financial relationships that could be construed as a potential conflict of interest.

## Footnotes

1 http://www.ncbi.nlm.nih.gov/geo

2 http://string-db.org

3 https://icgc.org/

4 https://portal.gdc.cancer.gov/

5 https://www.cbioportal.org/

6 https://david.ncifcrf.gov/home.jsp

7 https://www.kegg.jp/

8 http://software.broadinstitute.org/gsea/index.jsp

9 https://tcia.at/home

10 http://gepia.cancer-pku.cn/

## Abbreviations

HCC: hepatocellular carcinoma
GEO: Gene Expression Omnibus
DEGs: differentially expressed genes
PPI: protein–protein interactions
KEGG: Kyoto Encyclopedia of Genes and Genomes
String: Search Tool for the Retrieval of Interacting Genes
TCGA: The Cancer Genome Atlas
NUSAP1: nucleolar and spindle-associated protein 1
OS: overall survival.
